# Advanced Imaging Techniques in Skull Base Osteomyelitis Due to Malignant Otitis Externa

**DOI:** 10.1007/s40134-018-0263-y

**Published:** 2018-01-22

**Authors:** A. M. J. L. van Kroonenburgh, W. L. van der Meer, R. J. P. Bothof, M. van Tilburg, J. van Tongeren, A. A. Postma

**Affiliations:** 1 Department of Radiology, Maastricht University Medical Center, P. Debyelaan 25, 6229 HX Maastricht, The Netherlands; 2 Department of Anesthesiology, Maastricht University Medical Center, P. Debyelaan 25, 6229 HX Maastricht, The Netherlands; 3 Department of Otorhinolaryngology and Head and Neck Surgery, Maastricht University Medical Center, P. Debyelaan 25, 6229 HX Maastricht, The Netherlands

**Keywords:** Skull base osteomyelitis, Malignant otitis externa, CT, MRI, PET-CT, PET-MRI

## Abstract

**Purpose of Review:**

To give an up-to-date overview of the strengths and weaknesses of current imaging modalities in diagnosis and follow-up of skull base osteomyelitis (SBO).

**Recent Findings:**

CT and MRI are both used for anatomical imaging, and nuclear techniques aid in functional process imaging. Hybrid techniques PET-CT and PET-MRI are the newest modalities which combine imaging strengths.

**Summary:**

No single modality is able to address the scope of SBO. A combination of functional and anatomical imaging is needed, in the case of newly suspected SBO we suggest the use of PET-MRI (T1, T2, T1-FS-GADO, DWI) and separate HRCT for diagnosis and follow-up.

## Introduction

Malignant external otitis (MOE), also referred to as skull base osteomyelitis (SBO) or necrotizing otitis externa (NOE) is a serious condition which can be life-threatening [1•, 2••, 3, 4]. Usually, it is a complication of otitis externa when persistent infection fail to resolve with topical medications and aural toilet and the disease expands to the surrounding tissues [5]. Whereas MOE primarily affects the temporal bone, central or atypical SBO can be seen affecting the sphenoid and occipital bone, often centred on the clivus, and can be considered a variant of MOE [5].

Lesser et al. described three types of central skull base osteomyelitis [6]:

1. Necrotising otitis externa extending to the central skull base.
2. Central skull base osteomyelitis that presents after resolution of necrotising otitis externa.
3. Central skull base osteomyelitis as a primary presentation.

It most frequently presents as an extension of NOE; however, cases also can present without any clear proceeding lateral infection, making the diagnosis a difficult diagnostic challenge [6]. The disease was first described as a disease entity by Meltzer in 1959 and formally clinically defined by Chandler in 1968 [7, 8].

Patients can present with subtle and non-specific symptoms as persistent headache and eventual development of cranial neuropathy [9•]. However, most patients present with unrelenting otalgia that is disproportional to the clinical signs. There can be persistent purulent otorrhea, with an intact tympanic membrane and usually intact hearing. Otological examination may reveal oedema of the external auditory canal (EAC) and the presence of granulation tissue or polyp of the EAC floor near the junction of the osseous and cartilaginous portions [1•, 10]. The granulation tissue can be caused by the underlying osteitis. Patients can present with cranial nerve deficits, which can be caused by necrosis, neurotoxins and compression. Non-responsiveness to therapy is considered an important criterion in literature for the diagnosis of MOE [10].

Classically, SBO mostly presents in elderly patients with diabetes (85–90% of reported cases in literature), but younger patients may be susceptible to SBO when their immune system is compromised, for instance due to chemotherapy, HIV/AIDS or malnutrition [3]. Also, a recent study using the Nationwide Inpatient Sample database only found an incidence of 22,7% elderly diabetic patients in a retrospective cohort of 8300 SBO patients [11].

*Pseudomonas aeruginosa* is involved in a high percentage of all cases of SBO (50–90%), with a minor role for other bacterial agents, such as staphylococcal species, *Klebsiella*, *Proteus mirabilis*. In case of fungal pathogens, *Aspergillus fumigatus* is frequently encountered [2••].

## Pathophysiology

NOE is an invasive infectious disease involving the cartilaginous and/or the bony external canal giving rise to itching, otalgia, and/or otorrhoea. The (bacterial) infection causes bony erosions and uses fascial planes and venous sinuses for distant tissue invasion. It then can progress and spread to the surrounding osseous and soft tissues with involvement of the skull base and surrounding soft tissues, causing cranial nerve palsy and intracranial involvement.

The spread along the temporal bone through the fissures of Santorini commonly involves the styloid mastoid foramen (containing the facial nerve) and the jugular foramen (containing the glossopharyngeal, vagal and accessory nerves) [3]. Subtemporal extension starts at the osteocartilaginous junction, near the fissure of Santorini and spreads to the retrocondylar fat, the parapharyngeal fat, temporomandibular joint and masticator muscles.

Kwon et al. described four spreading patterns of the soft tissue extension [12•]: medial, anterior, crossed and intracranial spreading. In anterior spreading, there is extension and involvement of the masticator space and/or condylar bone marrow infiltration. In medial pattern, there is ipsilateral lateral nasopharyngeal wall thickening and/or ipsilateral preclival soft tissue infiltration. In the crossed pattern, the contralateral lateral nasopharyngeal wall is thickened with contralateral preclival soft tissue infiltration. When dural enhancement is present in the intracranial compartment, this is noted as intracranial extension [12•]. In addition to the above-mentioned patterns there can be intravascular involvement. Fungal spread is often intravascular and can leave the temporal bone relatively intact [2••].

**Fig. 1:**
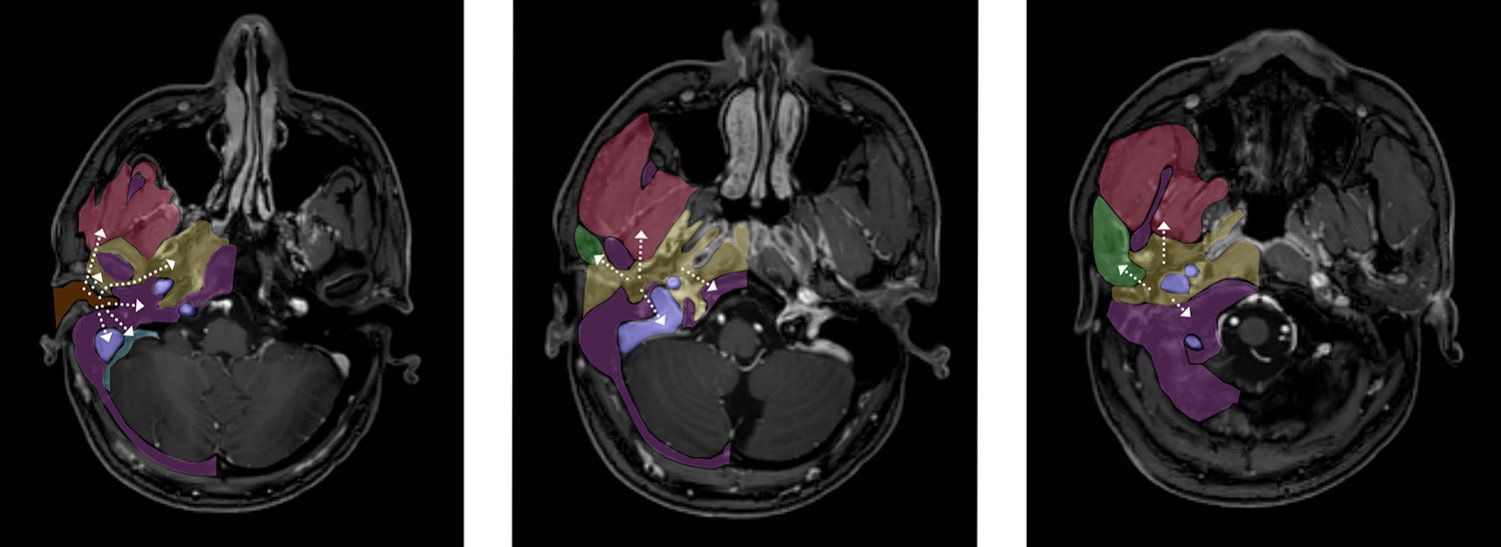
Spreading patterns. This figure illustrates the routes of infectious spread after NOE (EAC brown). After passing the fissures of Santorini, the infection can spread anteriorly to the fatty tissue (yellow) at the site of the temporomandibular joint and to the masticator space (red) and parotid gland (green). Medial route of spread entails the ipsilateral paranasopharyngeal fatty tissue where encasement of the internal carotid artery in the infectious site can occur (see also Fig. 5) and preclival soft tissue. From there, the infection is able to spread to the contralateral side (see also Fig. 3). Further extension through the osseous structures (purple) can lead to venous sinus thrombosis (see also Fig. 5) and dural extension (bright blue) (not shown in SBO cases but illustrated in the malignancy case in Fig. 2). Posteriorly, the infection can spread into the mastoid portion of the petrous bone (Color figure online)

In Table 1 and Fig. 1, a summary of spreading patterns and involved tissues is given.

**Table 1:** Spreading patterns of skull base osteomyelitis differentiated in compartments with the associated soft tissues and bone structures

Spreading patterns	Tissue involvement	
	Soft tissue	Bone and joint tissue
Anterior	Retrocondylar fat Masticator space and muscles Parotid gland Facial nerve	Temporal fossa Temporomandibular joint Styloid foramen
Posterior	–	Mastoid process
Medial/crossed	Parapharygeal fat Nasopharyngeal muscles and wall Glossopharyngeal nerve Vagal nerve Accessory nerve	Sphenoid Clivus Petrous apex Jugular foramen
Intracranial	Sigmoid sinus Jugular vein Internal carotid artery Dura	Jugular fossa Petroclival synchondrosis

### Diagnosis and Differential Diagnosis

The diagnostic approach of skull base osteomyelitis can be a challenging. As mentioned before, the spreading pattern can be diverse and can be non-specific for an infectious process. Advanced SBO can present with cranial nerve involvement, such as difficulty with swallowing and facial nerve palsy, thereby mimicking a neoplastic processes of the skull base. Clinicians should be careful to dismiss SBO based on the absence of infectious symptoms (fever, pain, swelling) and laboratory findings (increased ESR and white blood cell count), as this can be absent in immune-compromised patients [9•].

**Fig. 2:**
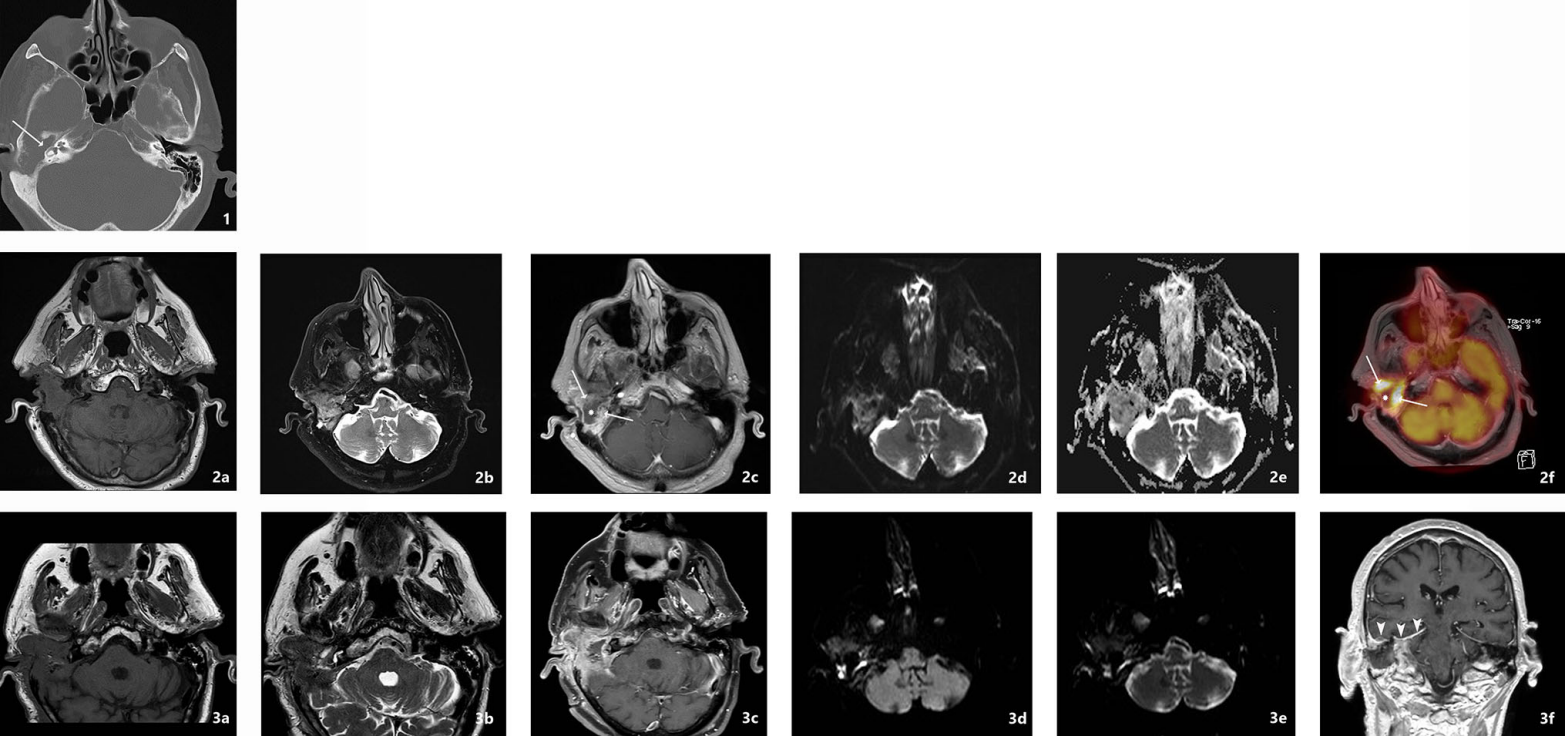
Differential diagnosis PCC of the EAC. A 84-year-old male patient presented with ongoing otalgia and otorrhea. His medical history showed two previous operations of the temporal bone. Prior to imaging the patient was treated with antibiotics and received a tissue biopsy which came out as negative. The patient did not have type 2 diabetes nor did he have a compromised immune system otherwise. The resection specimen eventually revealed a PCC malignancy. On CT bone erosion is seen as well as the soft tissue in the EAC (image 1). Furthermore, there is soft tissue in the inner ear (arrow). SBO tends to spare the tympanic membrane in contrary to malignant disease. MRI images show T1 hypointensity in the corresponding area. Contrast enhancement of the soft tissue is seen on the post-contrast T1-w scan (image 2c arrows). Interestingly, the central portion of the soft tissue component is hypointense (image 2c asterix) corresponding with the photopenic area on FDG-PET-MRI fusion images (image 2f asterix), this is probably due to central necrosis. In image 2d and 3d the diffusion restriction in this area can be appreciated. Follow-up scan after 6 weeks shows persistent contrast enhancement of the soft tissue and further dural enhancement (image 3f arrowheads)

Imaging findings for skull base osteomyelitis often show local tissue swelling and extensive diffuse bone destruction. However, the imaging appearance alone can be highly suggestive but non-specific for skull base osteomyelitis thus possibly delaying the diagnostic process. It is important to start treatment as soon as possible to prevent further infection spread and possible debilitating (intracranial) complications. On average, there is a diagnostic delay of 70 days between diagnosis and therapy reported in literature [10]. Neoplastic processes such as squamous cell carcinoma of the head and neck can also involve clival and preclival soft tissues and (focal) destruction the skull base (Fig. 2). Other differential imaging diagnoses are nasopharyngeal carcinoma, multiple myeloma, lymphoma, and metastatic processes [1•, 9•]. The specific imaging findings for skull base osteomyelitis are discussed in the section imaging modalities.

Thus, malignancy and SBO can mimic each other in clinical symptoms and imaging. Surgical tissue sampling of the affected tissue is often required and the only definitive method for discerning malignancy from an infectious process [2••]. One should be careful to dismiss a negative histologic biopsy material as a sampling error for malignancy. It is vital to realize that biopsy material is not only assessed for histology but for microbiological analysis as well to aid the choice in antibiotic treatment.

### Therapy

The mainstay therapeutic approach for skull base osteomyelitis is antimicrobial therapy. The mortality rate has improved significantly from 50 to 0–15% since the introduction of adequate antibiotic treatment with prolonged high-dose systemic antipseudomonal therapy [13]. The most commonly used antibiotic regime comprises ciprofloxacin augmented with an intravenous pseudomonas-sensitive beta-lactam antibiotic. One should be careful to declare a patient as successfully treated within the first year after treatment, as full disease eradication cannot be ascertained until after a year of symptom resolution because of frequent relapse of disease [4]. In the past, SBO was predominantly managed by means of surgical debridement. Surgical management is comprised of ear canal debridement, mastoidectomy, nerve decompression, or rarely dural plasty. Currently, the main role for surgical management is to discern infection from malignancy by taking bone biopsies. There is no consensus in current literature whether aggressive surgery, with or without leaving gentamycin containing soluble material, is a valid addition to antibiotic treatment. Also surgery can be a method as last resort in particularly hardy infectious spread when patients deteriorate under regular antimicrobial therapy [14].

### Prognosis

The prognosis of SBO has improved significantly with adequate antimicrobial therapy. Patients are more likely to have remitting disease or higher mortality rates show a more extensive infectious spread of SBO such as bilateral disease, involvement of the temporomandibular joint, infratemporal fossa or soft tissues of the nasopharynx [15]. The prognostic value of cranial nerve involvement such as the facial nerve remains unclear as some studies find an association with an increased mortality rate, while others dismiss this association [4, 15]. Overall, the survival for elderly patients is lower in comparison with younger patients, partially due to associated comorbidity. However, there is a higher morbidity rate than to be expected for the elderly with a significant age-related decline in survival. Patients above 70 year of age have a 5 year survival of 44%, in contrast to patients under 70 years with a 5-year survival rate of 75% [15].

### Diagnostic Challenges

Skull base osteomyelitis has proven to be a challenging disorder to diagnose. Its symptoms are often non-specific and signs of severe infection are mostly absent. As mentioned before, there is an important role for imaging in SBO diagnosis and investigating extent of disease spread, as well as monitoring treatment response and relapse rate. As lesions can consist of soft tissue abnormalities and bony erosions, as well as the dynamic process of inflammation, an optimal imaging modality has to be found. The goal of this review article is to give an up-to-date overview of the strength and weaknesses of current imaging modalities in diagnosis and follow-up of SBO. CT and MRI are used for anatomical imaging, whereas nuclear techniques aid in the functional process. Hybrid techniques such as PET-CT and more recently PET-MRI combine anatomical and functional biomarkers and bring imaging to a higher level.

## Imaging Modalities for SBO—Diagnosis

### Computerized Tomography

**Fig. 3:**
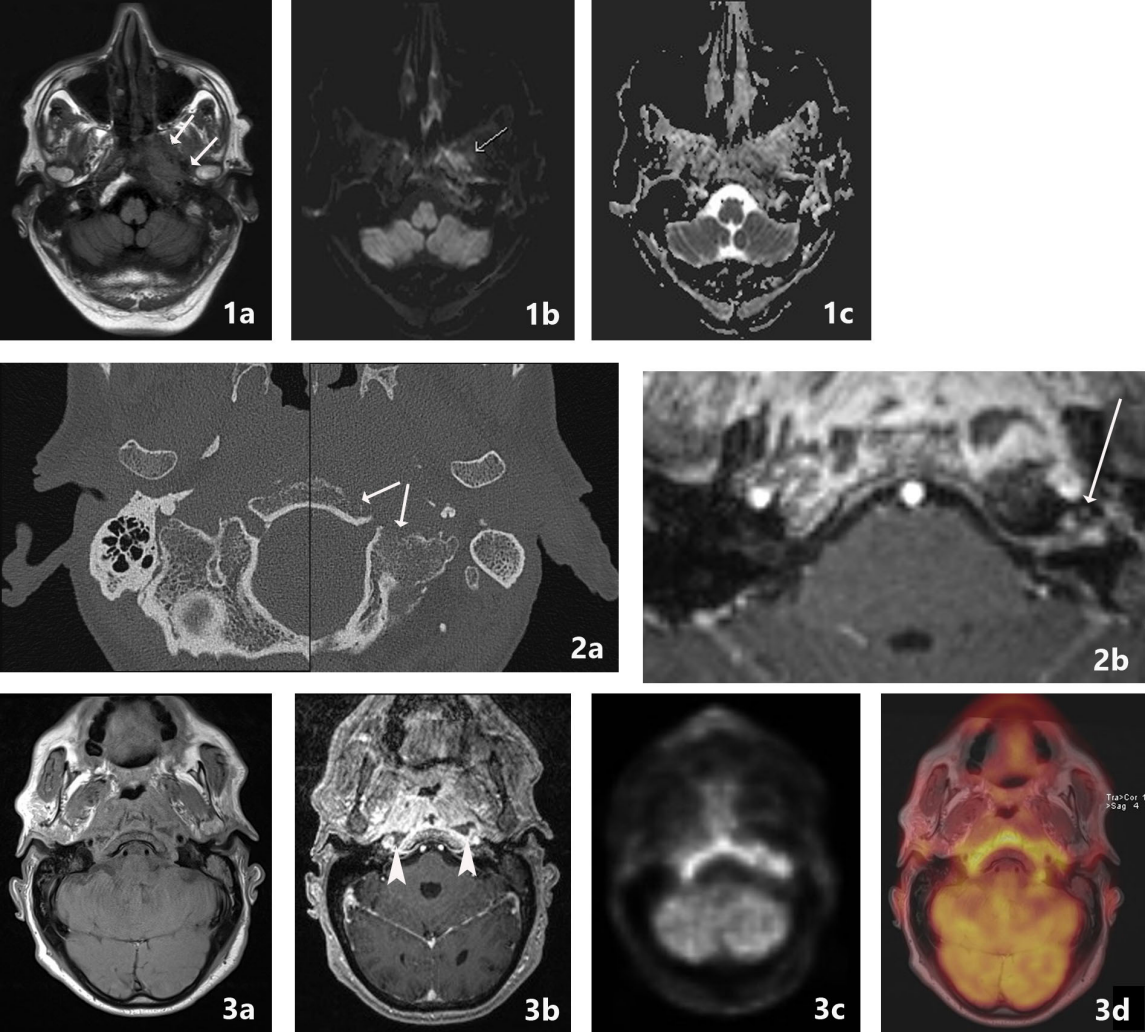
Abscess formation and involvement of facial nerve. A 65-year-old male with a history of a kidney transplant presented with headache and cranial nerve deficit, with dysphonia and facial nerve palsy. Otoscopy revealed a red and painful EAC. At MRI, there is SBO involvement of the bone marrow of the temporal bone and the clivus, with medial spreading pattern of the soft tissues (image 1a arrows). There is a hypointensity of the subtemporal soft tissues with obliteration of normal fatplanes in the masticator space. At DWI, a slight hyperintensity is shown (image 1b arrow), with relative low ADC (image 1c). At high-resolution CT demineralization and cortical destruction of the skull base and clivus can be appreciated (image 2a arrows). Follow-up PET-MRI (image 3a T1-w, 3b CE-T1-w) 1 month later shows SBO with expansion over the midline (crossed pattern) and abscess formation bilateral at the prevertebral region (image 3b arrowheads), with intense FDG avidity (image 3c PET, 3d colour fused PET-MR image). Involvement of the VII cranial nerve was seen at contrast-enhanced T1-w imaging, without osseous destruction of the facial canal (image 2b detail of skull base on T1-w-fs, arrow indicates enhancing facial nerve) (Color figure online)

**Fig. 4:**
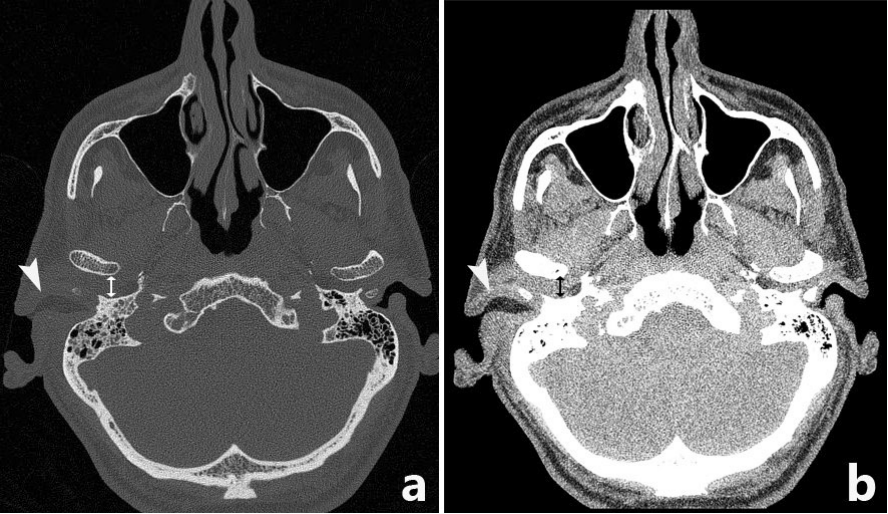
HRCT widening of temporomandibular joint. A 67-year-old male presented after 5 weeks with signs of external otitis and pain during chewing, without improvement after admission of topical antibiotics. An EAC polyp was removed. Bacterial culture revealed *Pseudomonas aeruginosa*. A non-contrast HRCT of the temporal bone was performed. The figure shows a bone window (image a) and soft tissue window (image b). Non-contrast-enhanced CT shows thickening of soft tissue of the EAC (image b arrow) as well as an anteriorly displacement of the mandible head with widening of the temporomandibular joint (black double arrow), indicating anterior spread of inflammation to the TMJ. At this point, no signs of bone erosion were present. Symptoms resolved after 4 weeks of iv. antibiotic therapy

Computerized tomography (CT) is the most commonly used imaging modality for diagnosis and follow-up by ENT clinicians for skull base osteomyelitis [16]. CT imaging is often viewed as a relatively easy and fast method to acquire an overview of the mastoid region. The strength of this modality is the evaluation of bone erosion and demineralization, especially with the use of (ultra)thin high-resolution CT in multiple planes [Fig. 3(2a)]. Skull base osteomyelitis is often caused by malignant otitis externa (MOE), although some cases have been described starting from the paranasal sinus due to acute-on-chronic sinusitis [9•]. Typical findings due to MOE is swelling of the external ear canal near the fissures of Santorini during physical examination [9•] (Fig. 4). CT imaging during this early stage will thus be non-specific as it shows soft tissue swelling with thinning of the fat planes (Fig. 4b). Detection of subtle changes can be improved by comparing the affected side with the contralateral one, although one must be careful to miss bilateral SBO cases. The spread for the external canal to the anterior wall (and thus posterior wall of the temporal mandibular joint) will show erosive changes of osseous structures. It is important to take note that skull base osteomyelitis does not always show osseous destruction in an early stage. The destruction of bone by itself is relatively a late phenomenon and in case of angioinvasion (fungal infections) changes in bone structure occur even later [17].

**Fig. 5:**
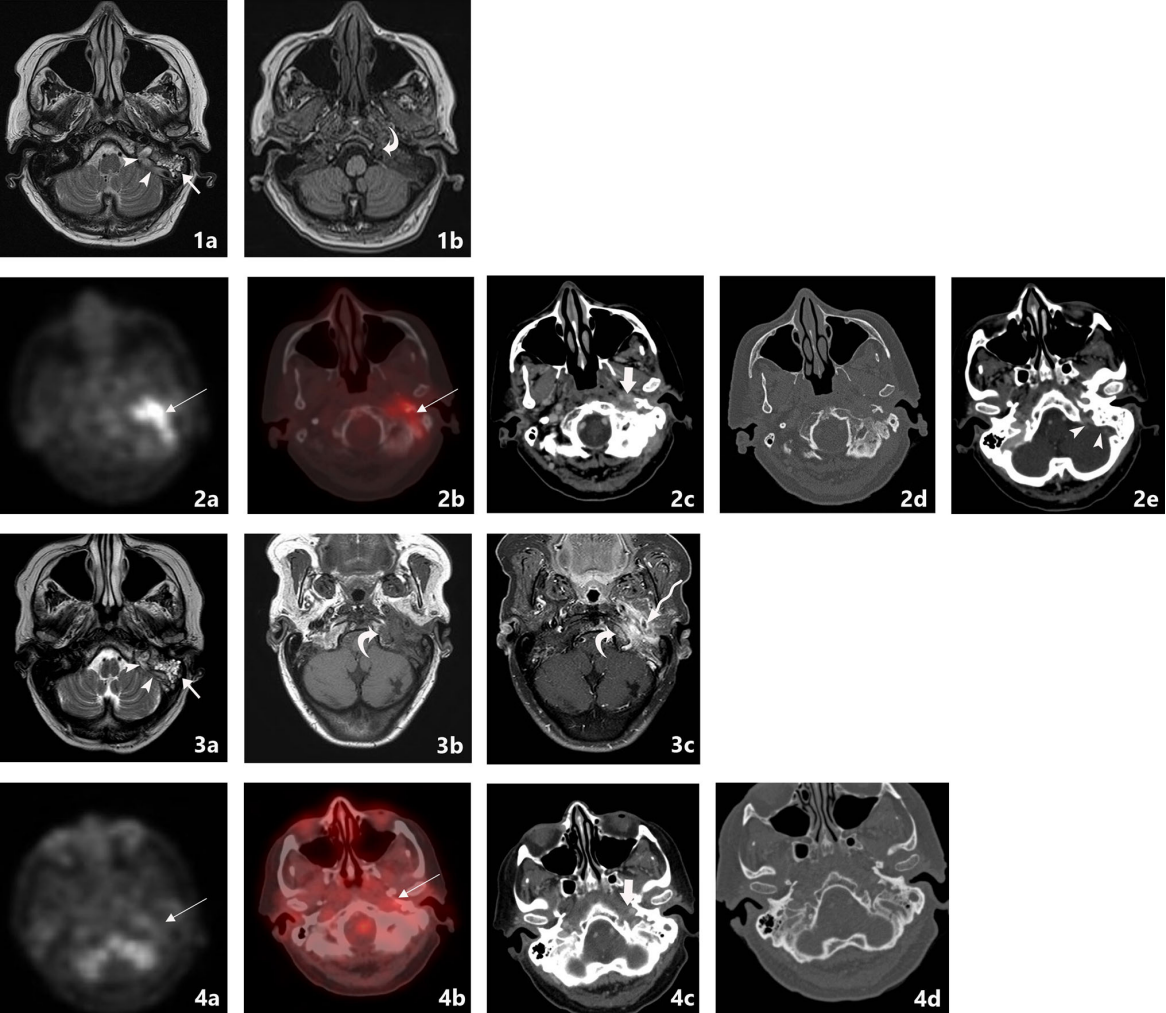
Transverse sinus thrombosis. A 76-year-old female presented with pain and otorrhea of the left ear since 2 months, with hearing loss and tinnitus. The symptoms were accompanied by a left-sided headache. No cranial nerve deficit was present. No history of diabetes or immune suppressive disease was present. Culture from the EAC revealed *Pseudomonas* as causal agent. An MRI was made at presentation with T2-w (image 1a) and T1-w (image 1b) series. At T2-w (image 1a) high signal intensity is present in the mastoid air cells at the left side (straight arrow), with loss of flow voids of the sigmoid sinus (arrowheads) indicative for sinus thrombosis. T1-w images show loss of signal intensity of the bone marrow of the skull base (image 1b, bent arrow), consistent with SBO. FDG-PET-CT performed 3 weeks after initial diagnosis confirms the location of SBO with increased uptake at the left temporal bone and surrounding soft tissue (image 2a, 2b fused PET-CT image, thin arrow). On the diagnostic CT, the decrease of subtemporal fatplanes with enhancing soft tissue at the stylomastoid foramen (image 2c thick arrow), bone erosion (image 2d) and sinus thrombosis (image 2e arrowheads) are reaffirmed. Additional MRI sequences (image 3a:T2-w; 3b:T1-w, 3c: T1-w fs) were executed showing added value of T1-w fatsat (fs) post-gadolineum scan (image 3c); the encasement of the internal carotid artery (arrow) is more easily appreciated within the area of extensive bone involvement (bent arrow). Follo-up FDG-PET-CT at 4 months (image 4a: PET; 4b: colour fused PET-CT; 4c: CE-CT soft tissue window; 4d: CE-CT-CT bone window) shows normalization of FDG avidity (image 4a thin arrow), normalization of enhancement of soft tissues (image 4b thin arrow) and sclerotic healing of the affected osseous tissue (image 4c thick arrow) (Color figure online)

The most frequent direction of SBO (80%) is expansion through the temporal bone with destruction of the temporomandibular joint, and erosion of the clivus [2••, 6, 18] [Fig. 5(2c, d)]. Involvement of the middle ear warrants malignancy as differential diagnosis, but can be present in SBO. The skull base foramina should be checked for irregularity and demineralization. The jugular foramen, the stylomastoid foramen, the lacerum foramen are frequently involved.

Next to the demineralization, soft tissue involvement is an important finding at CT. One should scrutinize the fat planes. The adagium “fat is your friend” is especially true in SBO. Subtle involvement of the soft tissues at CT can initially only be detected because of obliteration of normal fat planes (Fig. 4b), as are the retromandibular fat planes, the fat planes in the masticator space and the parapharyngeal space. More subtle fatplanes are at the styloid foramen and in the subtemporal region. It, therefore, seems logical that, next to the bone (high-resolution) kernels, soft tissue kernels should be calculated for optimal interpretation of the soft tissues (Fig. 4b).

It is important to realize that infectious spread of SBO does not occur in a standard pattern, and the understanding and recognition of anatomical structures is vital to recognize possible routes and the associated (intracranial) complications (Fig. 1). The medial spread to the nasopharynx occurs via the Eustachian tube, as it connects the middle ear and the nasopharynx. This will be visible as decreased fat planes in the subtemporal region and parapharyngeal space, soft tissue swelling and sometimes enhancement, in case iodinated contrast is given.

As stated above, the skull base foramina are frequently involved. Besides demineralization, it can be hard to detect abnormalities in these regions at CT. Clinical signs can be helpful to draw attention to the stylomastoid foramen in case of facial paralysis, whereas involvement of the foramen lacerum will not result in early clinical signs. Thereby, the spread to the foramen lacerum is an infectious passageway to intracranial involvement. Whereas CT is superior in visualization of bone, and of moderate reliability in soft tissues of the neck, intracranial evaluation is not a stronghold of CT imaging, thus any suspected involvement warrants further assessment by MRI imaging.

### Magnetic Resonance Imaging (MRI)

MRI is the superior imaging technique to evaluate the exact anatomical location and extent of the soft tissue components of SBO. In comparison with CT, there is higher soft tissue detail and resolution. Complications of SBO such as thrombosis and intracranial spread can be adequately assessed. Also, MRI scanning does not induce a radiation burden for the patient. We will now discuss the individual scanning sequences to understand the added value of each sequence and the information that can be obtained from each sequence.

#### T1-Weighted Sequences (T1-w)

AT T1-w images, fat has a high signal intensity. In SBO the normal fatty bone marrow of the skull base and temporal bone is replaced by inflammatory tissue. This results in decreased signal intensity at T1-w images without fat suppression [19] [Fig. 5(1b, 3b)].

The high signal intensity of the fat at T1-w is helpful for easy identification of the fat planes, and especially in case of SBO of involvement of the fat planes around the skull base, the subtemporal region, the masticator space, and the fat containing skull base foramina, e.g. the stylomastoid foramen [1•, 6, 9•]. These findings should alert you to look for SBO [Fig. 3(1a)]. The affected soft tissues and muscles are thickened and demonstrate amore hypointense signal on T1-w images.

Vascular encasement and involvement of the internal carotid artery and jugular vein can be assesed on T1-w images [9•] [Fig. 5(3c)]. Special attention should be paid to flowvoids on all sequences.

#### T2-Weighted Sequences (T2-w)

**Fig. 6:**
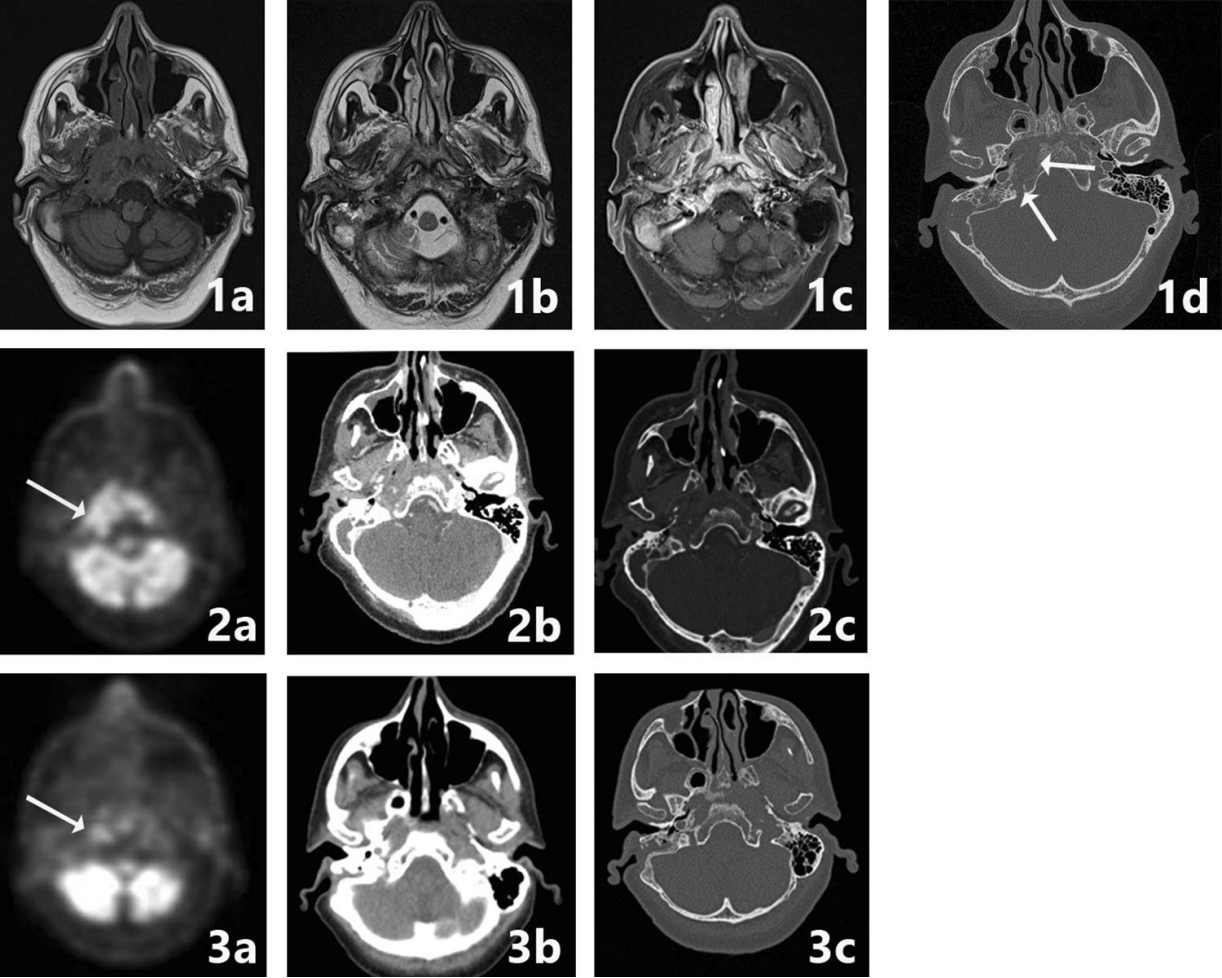
SBO crossed extension. A 64-year-old patient presented with clinical signs of a mastoid abscess. After initial successful surgery and antibiotic therapy, the patient presented 4 weeks later with progressive cranial deficit of the X and XI nerve. Imaging at that time was performed with CT and MRI in a referring hospital, with PET-CT imaging at 1 month (image 2a–c) and at 6 months follow-up. MRI with T1-w, T2-w, contrast-enhanced T1w-fs images (image 1a, b, c) and CT (image 1d) are shown. There is marked hypointensity at the bone marrow of the right skull base, the clivus and to a lesser extent of the left skull base, with involvement of the subtemporal soft tissues, the masticator space at right side, the prevertebral tissues and nasopharyngeal wall, with crossed extension to the left side. T1 FS post-gadolineum scan illustrates the extent of bone and soft tissue involvement (image 1c). Additional high-resolution CT shows bone erosion in the corresponding region, especially at the medial part of the skull base, the jugular foramen and the clivus (image 1d arrows). FDG-PET-CT indicates the metabolic activity and spread of the inflammation sites (image 2a arrows) and was used for follow up with a normalization of the FDG avidity after 6 months (image 3a arrows). The healing process is to a lesser extent seen on CT, but there is healing and remodeling of the cortical borders of the clivus (image 3c)

The affected area as suspected on T1-w images will, most of the time, be hypointense on T2-w images in contradiction with most infectious processes where T2-w images demonstrate a hyperintense signal because of hyperaemia and oedema [Fig. 6(1b)]. This hypointense aspect is thought to be due to the compromised vascular state in the (mostly diabetic) patients. The compromised vascular state may result in a reduced inflammatory response in comparison with normal infectious processes [1•]. T2-w images can be used to evaluate the flow voids in the vascular structures near the site of SBO. This way internal carotid artery occlusion and thrombotic changes in the jugular vein, sigmoidal and transverse sinus can be suspected and diagnosed [Fig. 5(1a, 3a)]. In case of suspicion of thrombosis, an additional magnetic resonance venography (MRV) might be helpful in evaluating the extent of the thrombosis. The same would be true for magnatic resonance angiography (MRA) in suspected carotic artery occlusion. Thus far, it is not standard protocol to add MRA or MRV sequences in the evaluation of SBO.

#### Post-Gadolineum T1-Weighted Sequences

After admission of Gadolineum contrast medium, the involved skull base and soft tissues will enhance diffusely [Figs. 3(3b), 5(3c), 6(1c)]. This T1-w post-contrast scan is best performed in combination with fat suppression, to correctly interpret the extent of enhancement and differentiation from the otherwise hyperintense fatty bone marrow and soft tissues [compare Fig. 2(2c–3c)]. Next to osseous involvement, one should pay attention to the posterior and middle fossa to look for dural enhancement and intracerebral extension of the infection [1•, 9•, 12•] [Fig. 2(3f)].

At this moment, there are no definite imaging characteristics that can differentiate between malignant disease and SBO. Several possible MRI characteristics can aid in the differential diagnosis. For instance in comparison with malignant disease of the skull base, SBO will respect the anatomical fascial planes. This can be best appreciated after admission of Gadolineum contrast medium [6]. Another possible sequence that can aid in this respect is diffusion-weighted imaging.

#### Diffusion-Weighted Imaging (DWI)

Diffusion-weighted imaging can be helpful in SBO [Fig. 3(1b, c)] especially aiding in the differentiation between lymphoma/nasopharynx carcinoma and bacterial SBO [17]. Apparent diffusion coefficient (ADC) values in malignant disease tend to be reduced because of reduced extracellular matrix, enlargement of the nuclei and hypercellularity of malignant disease [20]. Bacterial SBO on the other hand shows higher values of ADC. However, restricted diffusion, increased signal on DWI, with low ADC should make you beware of associated abscess formation in SBO [21]. The main difficulty of diffusion-weighted images in the skull base region is the susceptibility artefacts arising from the inhomogeneous tissues in this region. The use of non-epi DWI imaging, as is used in cholesteatoma imaging, can be helpful to overcome these artefacts [22].

#### Advanced Techniques

Until thus far, no role has been described for functional MRI imaging techniques such as MRI perfusion of MRI spectroscopy. One could speculate that this could aid in differential diagnosis, but more research is needed before drawing any conclusion.

MRI imaging thus shows the extension and involvement of the skull base, its foramina and the vessels and nerves at high resolution and is, therefore, very important to describe and delineate the anatomical extension of the soft tissue involvement of the SBO. The osseous involvement can be detected by the abnormal bone marrow signal, but cortical abnormalities remain undetected. Since complaints often are present for several weeks to months, active inflammation is difficult to discriminate from resolving infection.

## Nuclear Imaging

Tracers in nuclear medicine are used for functional imaging rather than anatomical imaging as in CT and MRI. They can be divided in gamma tracers and beta-emitting tracers. They all do not provide a single solution to the diagnostic challenge in skull base osteomyelitis thus far, but can aid in initial diagnosis or disease followup. We will discuss the working mechanism of all the different tracers with the advantages and disadvantages of each technique as well as future perspectives.

### Gamma Tracers

Tc-99 m-MDP correlates with increased osteoblastic activity. Even a 10% rise in osteoblastic activity can be detected [23]. The scan is positive early in the process of osteomyelitis. It is a cheap and easily available technique. On the down side, it will also show increased uptake in other conditions with a high bone turnover, for instance post-operative state or malignancy with bone involvement [1•]. Changes lag behind clinical improvement, therefore it cannot be adequately used in follow-up of treatment response [1•]. In general, three-phased bone scintigraphy studies are used in assessing infectious disease. The arterial blood flow and venous blood pool phase show an increased tracer activity corresponding with increased vascular supply in infectious disease due to capillary dilatation.

Technetium-labelled leucocytes are able to detect infectious foci but are less commonly used since it is more expensive and more laborious in comparison with other techniques [24]. It is less able to detect low-grade infections [25]. Other disadvantages are the possibility of non-specific accumulation of leucocytes in normal bone marrow and the high blood pool activity.

Gallium-67-Citrate binds to actively dividing cells, for instance leucocytes in infectious processes [1•, 23]. It accumulates in soft tissue and bone infections. Papers discussing this tracer refer to an article published in 1985 explaining the normalization of Ga-67-citrate scans concurrent with treatment response. Since adequately treated infectious lesions lose the ability to concentrate the tracer. Therefore, these traces are often used in treatment response monitoring [26]. Disadvantages are the high costs, time consumption, and high radiation dose [25, 27].

All gamma tracers tend to have low spatial resolution and lack anatomical detail. By means of single-photon emission tomography computed tomography (SPECT-CT), this is evidently improved. This technique combines 3D tracer imaging with the higher resolution of CT, improving anatomical correlation [28, 29]. Spatial resolution on the other hand can be improved by the use of beta emitters. This technique is based on the principle of coincidence detection, which greatly improves the spatial resolution in comparison with conventional nuclear imaging.

### Beta-Emitting Tracers

**Fig. 7:**
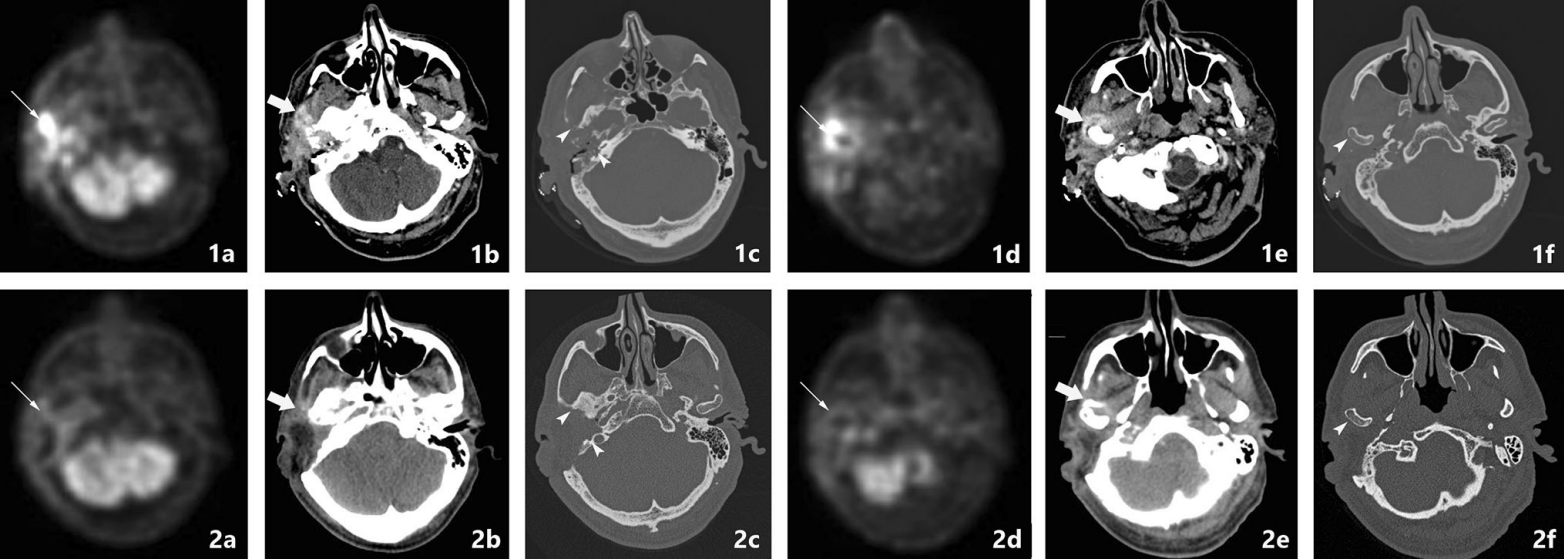
PET-CT follow-up. A 63-year-old male presented pain and otorrhea of the right ear for 3 months. Clinical symptoms included hearing loss and signs of vertigo. He was diagnosed with NOE. Initial FDG-PET-CT scan shows increased FDG uptake in the soft tissue in the subtemporal region expanding to the masticator space (thin arrow in image 1a) and the temporomandibular joint (thin arrow in image 1d). Corresponding CT in soft tissue setting diminishing of the fatplanes of the retromandibular space and masticator space, with enhancement of the peri-mandibular region and parotid region, corresponding with infectious spread (image 1b, 1e thick arrows). CT in bone window shows bone erosion of the temporal bone and mandibular condyle (image 1c,1f arrowheads). The patient received antibiotic therapy and a lateral temporal bone resection. Follow-up FDG-PET-CT scan shows markedly decreased FDG avidity (image 2a, 2d thin arrows), with decreased enhancement of soft tissue (image 2b, 2e thick arrows) as well as sclerotic margins of involved bone segments (image 2c, 2f arrowheads)

**Fig. 8:**
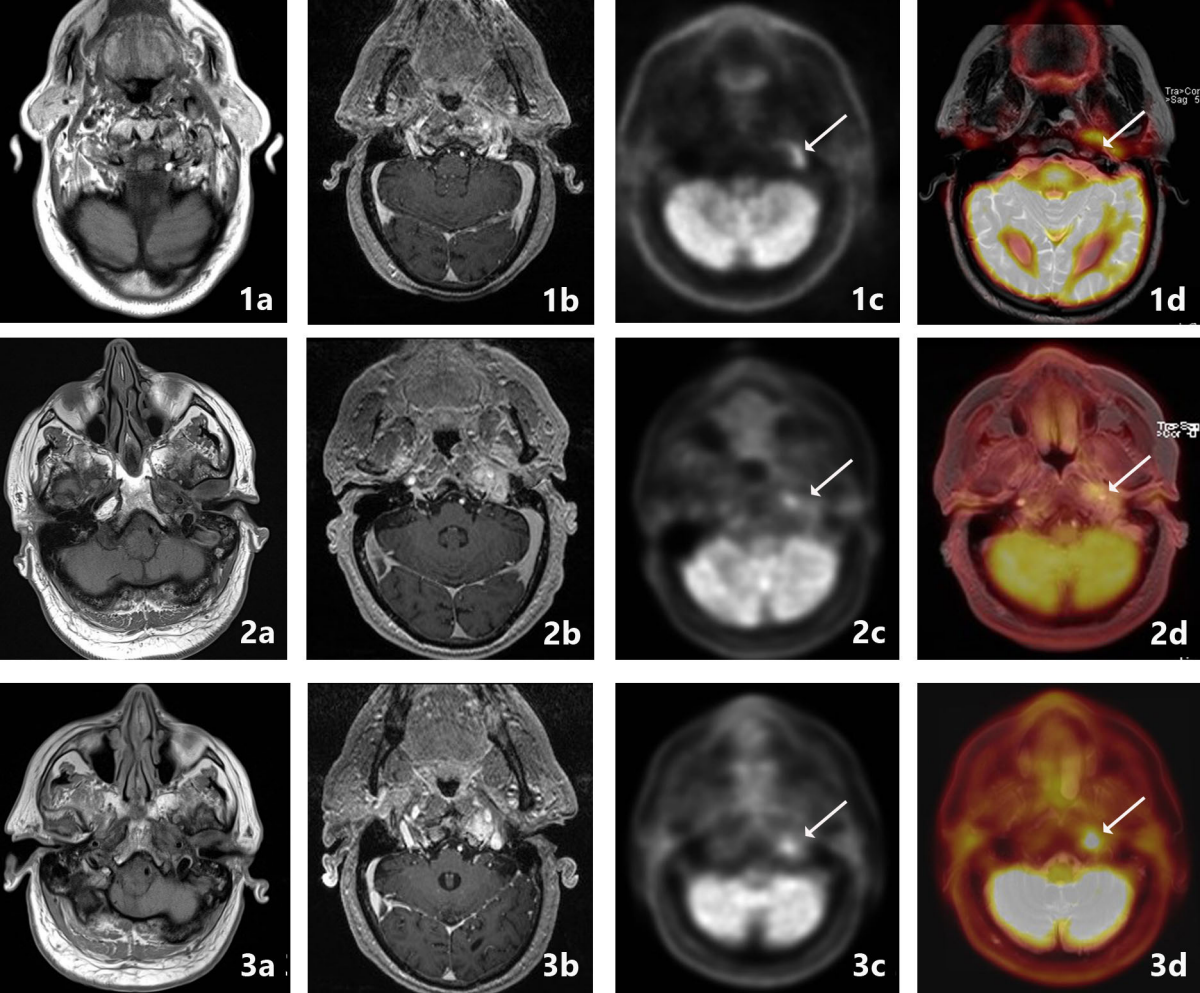
PET-MRI follow-up. A 85-year-old male, with a history of diabetes, presented at a referring hospital 3 months earlier. He was treated with topical and intravenous antibiotics for external otitis. After initial improvement of symptoms, the patient presented with facial nerve palsy and recurrent pain with continuous hearing loss. At CT (not shown) SBO was suspected with obliteration of subtemporal fat planes, induration of the fat at the stylomastoid foramen, osseous destruction of the ventral border of the mesotympanum and malleus. Follow-up was performed with FDG-PET-MRI and additional CT. No increase of bone erosion on CT was noted (not shown). PET-MRI images are shown at 2, 5 and 7 months’ follow-up (respectively, upper, middle and lower panels). From left to right T1-w, contrast-enhanced T1-w, PET and colour fused PET-MRI images are shown. A hypointense signal intensity can be appreciated on the T1-w images (image 2a, 3a, 4a), with loss of fat planes and enhancement after administration of gadolinium (image 2b, 3b, 4b). There is increased uptake of FDG in the temporal bone and subtemporal region, as well as in the retromandibular region (arrows). At follow-up, MRI abnormalities resolved at the meatus and mandibular region with persistent signal abnormalities and enhancement at the medial part of the skull base. The uptake at and below the skull base is decreased at the 5 month follow up, whereas at 7 months there is again an increase in FDG uptake, with new bone marrow edema on T2-w images at the clivus (not shown). Again iv. antibiotics were given; at follow-up at 9 months there was clear improvement in FDG uptake and bone marrow edema. Interestingly enough the increased FDG avidity at the 7-month interval scan (image 3d, 3e) is hardly appreciated on the given images (the scaling of the images was not intentionally adjusted for publication). Showing the importance of quantitative measurement of FDG avidity and comparison with the contralateral side (in accordance with the research of Wong et al.) (Color figure online)

The main beta emitter used in infectious disease is FDG (2-Fluor-2-Desoxy-Glucose). This glucose analogue is able to detect increased metabolism in case of an infection. Other conditions such as malignancy also increase metabolism, therefore, FDG is not a specific infection tracer. It can, however, aid in deterring disease extent and evaluation of treatment response (Figs. 5, 6, 7 and 8). Its widespread clinically availability, good spatial resolution and decrease in radiation burden compared to Gallium make this the nuclear diagnostic tracer of first choice [27].

In osteomyelitis research not specifically aimed at the skull base another tracer has been suggested to give additional information on bone destruction and inflammation. Na-F (sodium fluoride) can detect increased bone turnover. In a three-phased scan this tracer might also be able to identify infectious foci [30]. Although there is very limited evidence, this is an interesting tracer to keep in mind.

We stress an objective quantitative measurement of tracer accumulation in all above-mentioned techniques in concordance with the work of Wong et al. [31] (Fig. 8). As well as comparison with the non-lesion side to be more sensitive in disease evaluation. A clear cut evidence-based cutoff value is still lacking, hopefully future research will provide this.

In concordance with the gamma tracers, beta-emitting tracers lack anatomical detail. This can be compensated with hybrid imaging techniques such as positron emission tomography-CT (PET-CT) or PET-MRI, which is a more recently described imaging technique.

Hybrid imaging with CT is advantageous to either correlate with bone resorption and to a lesser extend soft tissue/involvement [32, 33]. PET-MRI has recently become available. It combines the superior soft tissue contrast of MRI, as stated before, with simultaneous acquired functional imaging of PET. Since it combines functional and high-resolution anatomical imaging, it gives a more accurate opinion about the actual process of inflammation at the diverse anatomical subsites of the skull base and surrounding soft tissues, providing a dual modality biomarker.

Soft tissue detail in MRI is much higher than in CT as mentioned above. To obtain more information about soft tissue involvement on CT Iodine contrast medium is preferred, although MRI still is preferred. The radiation burden of PET-CT is significantly reduced by combining PET with MRI since MRI is not a X-ray-based technique. PET-scanning as well as MRI scanning are quite time consuming. By concurrent PET-MRI scanning this time frame is reduced. An additional high-resolution CT will only add several minutes to the diagnostic process. Contra-indications of PET-MRI scanning are similar to the known MRI contra-indications and entail severe claustrophobia and non-MRI-compatible metal implants. Examples of PET-CT hybrid imaging are seen in Figs. 5, 6 and 7. Examples of PET-MRI imaging are seen in Figs. 2, 3 and 8.

In general, we advocate the initial work-up and follow-up with PET-MRI, with PET-CT being the second best option in case of valid contra-indications for MRI scanning.

## Imaging Modalities for SBO—Follow-up

At present, there is no consensus about the preferred imaging method of SBO follow-up, as depicted by a survey involving 221 ENT specialists [16]. As is to be expected, all previous mentioned imaging methods have their own advantages and disadvantages.

Follow-up via CT scan is measured by bone remineralization, however, this does not immediately occur after disease resolution and it is a relative late phenomenon thus making CT a less reliable imaging modality to evaluate (early) treatment response [1•, 5, 13].

MRI imaging is known to be a reliable imaging modality to monitor soft tissue changes in the diagnosis of SBO, such as reduced fat planes or medullary bone abnormalities. Although abnormalities in soft tissues are easily depicted, resolution of abnormalities often lags behind disease resolution. Furthermore, an abnormal bone marrow signal can still be present for 6–12 months after successful treatment, making MRI unreliable for distinguishing resolved from ongoing SBO of the bone marrow [5, 34].

Functional imaging, such as nuclear imaging used for disease can be used for follow-up. Also in conventional nuclear imaging many of the diagnostically valuable changes stay present after the resolution of disease. As stated above, Technetium-99 m is indicative of osteoblastic activity in damaged bone [9•, 26]. Because of prolonged activities of osteoblasts in previously affected bone, Tc-99 m will continue to demonstrate previously affected areas as hot spots for many years [24, 33]. PET using fludeoxyglucose is better suited for monitoring disease resolution, as FDG shows ongoing neutrophil activity in metabolically active tissues, and decreased inflammation gives less signal intensity without much delay.

### Timing of Follow-up

SBO is a severe condition which warrants adequate regular clinical and radiological follow-up, especially during the first year after treatment. Follow-up is of importance to detect early SBO relapse and to monitor possible non- treatment response, as this could identify a misdiagnosed skull base malignancy (Fig. 2).

Proposed strategies range from imaging when new clinical symptoms present, to intermittent imaging every 6 weeks until no infectious process can be viewed [35]. The ultimate imaging technique should have a high resolution, be specific for signs of infection and should use the lowest possible achievable radiation dosage since imaging must often be repeated. Moreover, the imaging modality should be able to detect changes in soft tissues, bone marrow and subtle increases in bone turnover. With respect to all these characteristics, hybrid forms of imaging come closest to the ideal imaging modality portrayed above. The union of MRI and FDG-PET presents the best of both worlds; superb spatial resolution to detect subtle abnormalities in soft tissues and bone marrow, as well as strong evidence of altered bone metabolism, see Table 2 for an overall summery of imaging strengths. Since repetitive imaging is needed in SBO follow-up, the use of PET-MRI is also favoured as it reduces the amount of radiation exposure compared to PET-CT [36].

**Table 2:** Relative strengths and weaknesses of imaging modalities used for skull base osteomyelitis diagnosis and follow-up

Evaluation characteristics	Radiology		Nuclear	Hybrid	
	CT	MRI	SPECT (Tc-99 m- MDP)	FDG-PET/CT	FDG-PET/MRI
Bone erosion	++	−	−	+	−
(Bone) metabolism	−	−	+	+	+
Soft tissue	±	+	−	±	++
Spatial resolution	+	++	−	±	+
Radiation	−	+	−	−	±
Follow-up	−	−	−	±	+

PET-MRI is the most effective imaging method for proper diagnosis and follow-up

## Conclusion

The diagnostic challenge in osteomyelitis lies in the fact that no single modality is able to address the scope of the disease. A combination of functional and anatomical imaging is needed to fully understand the spread of disease and associated complications. High-resolution CT shows the extent of bone erosion. Soft tissue extension is best evaluated with MRI. FDG-PET can be used to assess the metabolic active disease and can be used in follow-up. Older techniques such as Tc-99 m-MDP and Ga67 scintigraphy must be performed as SPECT scan in combination with CT to have added diagnostic value. DWI MRI sequences and three-phased Na-F PET-CT are interesting imaging techniques but their strength have to be evaluated in further prospective research. Our suggestion in new cases suspect of SBO would be preferably a combination PET-MRI (T1, T2, T1-FS-GADO, DWI) and separate high-resolution CT or in case a hybrid PET-MRI scanner is not available at your hospital FDG-PET-(HR)CT and separate MRI (T1, T2, T1-FS-GADO, DWI). The combination of FDG-PET and either MRI or CT can then also be used for follow-up.

## Abbreviations

ADC: Apparent diffusion coefficient
CT: Computerized tomography
DWI: Diffusion-weighted imaging
EAC: External auditory canal
ESR: Erythrocyte sedimentation rate
Fs: Fat saturated
FDG: 2-Fluor-2-desoxy-glucose
Ga-67: Galium-67
MDP: Methyldiphosponate
MOE: Malignant otitis externa
MRA: Magnetic resonance angiography
MRI: Magnetic resonance imaging
MRV: Magnetic resonance venography
Na-F: Sodium fluoride
NOE: Necrotizing otitis externa
PET: Positron emission tomography
SBO: Skull base osteomyelitis
SPECT: Single-photon emission tomography
Tc-99 m: Technetium-99m
T1-w: T1-weighted sequences
T2-w: T2-weighted sequences
